# Relationships between gross motor skills, cardiovascular fitness, and visuospatial working memory-related brain activation in 8- to 10-year-old children

**DOI:** 10.3758/s13415-020-00805-5

**Published:** 2020-07-22

**Authors:** Irene M. J. van der Fels, A. G. M. de Bruijn, R. J. Renken, M. Königs, A. Meijer, J. Oosterlaan, D. D. N. M. Kostons, C. Visscher, R. J. Bosker, J. Smith, E. Hartman

**Affiliations:** 1 Center for Human Movement Sciences, University Medical Center Groningen, University of Groningen, Postbus 196, 9700 AD Groningen, the Netherlands; 2 Groningen Institute for Educational Research, University of Groningen, Groningen, the Netherlands; 3 Neuroimaging Center Groningen, University Medical Center Groningen, University of Groningen, Groningen, the Netherlands; 4 Emma Children’s Hospital, Amsterdam UMC, University of Amsterdam and Vrije Universiteit Amsterdam, Emma Neuroscience Group, Department of Pediatrics, Amsterdam Reproduction & Development, Amsterdam, the Netherlands; 5 Clinical Neuropsychology Section, Vrije Universiteit, Amsterdam, the Netherlands

**Keywords:** Neuroimaging, Brain functioning, Physical fitness, Executive functions, Cognition

## Abstract

Relationships between gross motor skills and cardiovascular fitness with visuospatial working memory (VSWM) in children are hypothesized to be mediated by underlying functional brain mechanisms. Because there is little experimental evidence to support this mechanism, the present study was designed to investigate the relationships of gross motor skills and cardiovascular fitness with VSWM-related brain activation in 8- to 10-year-old children. Functional magnetic resonance imaging data obtained during a VSWM-task were analyzed for 80 children from grades 3 (47.5%) and 4 of 21 primary schools in the Netherlands (51.3% girls). Gross motor skills (Korper Koordinationstest für Kinder and Bruininks-Oseretsky Test of Motor Proficiency - 2nd Edition) and cardiovascular fitness (20-meter Shuttle Run Test) were assessed. VSWM-related brain activation was found in a network involving the angular gyrus, the superior parietal cortex, and the thalamus; deactivation was found in the inferior and middle temporal gyri. Although behavioral results showed significant relations of gross motor skills and cardiovascular fitness with VSWM performance, gross motor skills and cardiovascular fitness were not related to VSWM-related brain activation. Therefore, we could not confirm the hypothesis that brain activation underlies the relationship of gross motor skills and cardiovascular fitness with VSWM performance. Our results suggest that either the effects of physical activity on cognition do not necessarily go via changes in gross motor skills and/or cardiovascular fitness, or that brain activation patterns as measured with the blood-oxygen-level dependent (BOLD) signal may not be the mechanism underlying the relationships of gross motor skills and cardiovascular fitness with VSWM.

## Introduction

Gross motor skills represent the involvement of large body muscles in balance, limb, and trunk movements (Corbin, Pangrazi, & Franks, 2000). Gross motor skills that children acquire during childhood enable further development of complex movement and sport-specific skills (Clark & Metcalfe, 2002). Well-developed gross motor skills go hand in hand with higher levels of physical activity, which are important for developing higher levels of cardiovascular fitness (Clark & Metcalfe, 2002). Cardiovascular fitness refers to the ability of the circulatory and respiratory systems to supply oxygen during sustained physical activity (Corbin et al., 2000). Low cardiovascular fitness levels have shown to be related to cardiovascular disease risk factors, increased body fatness, and hypertension in children and adolescents (Ortega, Ruiz, Castillo, & Sjöström, 2008). Therefore, physical fitness is not only an important aspects for children’s physical development but also for their health (Ortega et al., 2008; Robinson et al., 2015; Stodden et al., 2008).

There is accumulating evidence that gross motor skills and cardiovascular fitness are related to executive functioning in children (Haapala, 2013; Van der Fels et al., 2015). Executive functioning refers to a subset of interrelated processes that are involved in purposeful, goal-directed behavior, including inhibition, working memory, and cognitive flexibility (Miyake et al., 2000; Banich, 2009; Diamond, 2013). Executive functions are important for success throughout life and play a critical role in the development of academic skills (Best, Miller, & Jones, 2009; Best, Miller, & Naglieri, 2011; Bull, Espy, & Wiebe, 2008). Underlying functional brain mechanisms are thought to be responsible for the relationships of gross motor skills and cardiovascular fitness with executive functions (Leisman, Moustafa, & Shaffir, 2016; Middleton & Strick, 2000). However, little direct evidence supports these underlying mechanisms (Ludyga et al., 2019; Chaddock et al., 2012; Voss et al., 2011). Therefore, this study was designed to get a better insight into the brain mechanisms underlying the relationship between physical variables and executive functions.

## Gross motor skills and visuospatial working memory

Behavioral studies have shown that gross motor skills are related to the executive functions that are most directly involved in gross motor tasks in children, such as visuospatial working memory (VSWM) (Rigoli, Piek, Kane, & Oosterlaan, 2012; van der Fels et al., 2019). VSWM refers to the ability to maintain and manipulate visuospatial information over brief periods of time (Baddeley & Hitch, 1994). VSWM is an important executive function, because it is a prerequisite for several cognitive processes, such as logical reasoning, problem solving, and academic performance (Baddeley & Hitch, 1974, 1994; Diamond, 2013).

In children and adults, functional neuroimaging studies have shown VWSM-related brain activity in frontal areas (van Ewijk et al., 2015; Kwon, Reiss, & Menon, 2002; Nelson et al., 2000), parietal areas (Kwon et al., 2002; van Ewijk et al., 2015; Nelson et al., 2000), the occipital cortex (Nelson et al., 2000; van Ewijk et al., 2015), the premotor cortex (Kwon et al., 2002), and in the cerebellum, the thalamus, and the insula (van Ewijk et al., 2015). Therefore, VSWM seems to be facilitated by a complex network of brain activity. It is hypothesized that the neural network involved in VSWM tasks also is important for the planning, execution, and control of movements, thereby explaining the relationship between gross motor skills and VSWM (Goldberg, 1985; Diamond, 2000; Dum & Strick, 1991; Künzle, 1978; Tanji, 1994; Wiesendanger, 1981).

There has been some support for the relationship between motor skills and VSWM-related brain activity. Using EEG, Ludyga et al. (2019) showed in a longitudinal study with 52 children, aged 8–10 years, that better motor skills at baseline were related to better attentional and preparatory processes during a working memory task 9 months later. This was mainly expressed in the premotor and motor cortex and in the frontoparietal network. Furthermore, Ludyga, Gerber, Kamijo, Brand, and Pühse (2018) investigated effects of an 8-week physical activity intervention (20 min each school day) that included aerobic activity and coordinative exercises on brain functioning during a visuospatial working memory task. The study showed enhanced brain functioning, which was expressed as an increase of the initial contingent negative variation (CNV) of event-related potentials, during visual working memory of adolescents mainly in the premotor and motor cortex and in the frontoparietal network (Ludyga et al., 2018). It is important to further investigate the relationship between gross motor skills and VSWM-related brain activity. By examining how gross motor skills relate to brain regions underlying VSWM in preadolescent children, using fMRI (having a higher spatial resolution than EEG), we hope to get a better idea of the exact location of brain regions that are important in the development of gross motor skills and VSWM.

## Cardiovascular fitness and visuospatial working memory

Not only gross motor skills, but also cardiovascular fitness, has shown to be related to VSWM (de Bruijn, Hartman, Kostons, Visscher, & Bosker, 2018; Scudder et al., 1988). To explain the relationship between cardiovascular fitness and VSWM, the cardiovascular fitness hypothesis has been brought forth. Participation in physical activity is assumed to lead to changes in the cardiovascular system (physical fitness), which go hand in hand with changes in the brain, such as increased cerebral blood flow and the up-regulation of neurotransmitters, which in the long term leads to neurogenesis and angiogenesis, in turn resulting in better cognitive performance on, amongst others, executive function tasks (Cotman, Berchtold, & Christie, 2007; Dishman et al., 2006; Sibley & Etnier, 2003).

There is some support for this hypothesis from neuroimaging studies, showing that cardiovascular fitness is related to neural networks supporting executive functioning. However, this evidence is mainly provided for inhibition. Chaddock et al. (2012) and Voss et al. (2011) have shown that 9- to 10-year-old children with higher cardiovascular fitness showed less frontal, parietal, and temporal inhibition-related brain activity, and this was related to higher levels of accuracy on the inhibition task. Furthermore, the relationship between cardiovascular fitness and memory performance have been shown to be mediated by brain structures. A study by Chaddock et al. (2012) showed relations between cardiovascular fitness and memory performance, and this relationship was mediated by greater hippocampal volume. We are not aware of studies investigating relationships between cardiovascular fitness and VSWM-related brain functioning. It is important to investigate this relationship, because VSWM is important for several cognitive processes and academic performance. Therefore, interventions to improve cardiovascular fitness may bring about functional changes in the brain that are important for VSWM, subsequently also resulting in positive effects on several other cognitive processes as well as academic achievement.

## The present study

The main goal of the present study was to investigate relationships of gross motor skills and cardiovascular fitness with VSWM-related brain activation in 8-to 10-year-old typically developing children. First, the pattern of VSWM-related brain activation will be examined. Subsequently, gross motor skills and cardiovascular fitness will be related to the observed VSWM-related activity patterns. To clarify the hypothesis that brain activity is the mechanism underlying the relationship of gross motor skills and cardiovascular fitness with VSWM, the relationship of both gross motor skills and cardiovascular fitness with behavioral VSWM performance during scanning also is reported. It is hypothesized that both gross motor skills and cardiovascular fitness will be associated with VSWM performance and VWSM-related brain activation. The results of this study will contribute to our understanding of the mechanisms underlying the relationship between physical capacities and VSWM, which will help in the development of physical activity interventions that also can stimulate brain development important for executive functioning.

## Materials and methods

### Participants

A total of 92 children from 21 schools in the Netherlands were included in this study (47 girls, 51.1%). Participating children were in grade 3 (*n* = 46, 50.0%) or grade 4 and were 8–10 years old (mean = 9.14 years, standard deviation [*SD*] = 0.63). This study was part of a large cluster randomized, controlled trial (RCT; “Learning by Moving”) that assessed the effects of two types of physical activity on cardiovascular fitness, gross motor skills, cognitive functions, and academic performance. Only baseline data of the RCT was used for the current study. Children who participated in the cluster RCT were invited to participate in this magnetic resonance imaging (MRI) sub-study. Only children older than age 8 years who had no contraindications for MRI were included. Written, informed consent was provided by children’s parents or legal guardians. This study was approved by the ethical board of the Vrije Universiteit Amsterdam (VCWE-S-15-00197) and registered in the Netherlands Trial Register (NL5194).

### Tasks

#### Visuospatial working memory during scanning

**Fig. 1:**
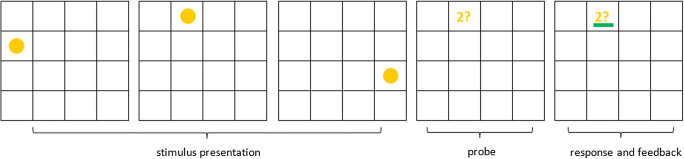
Schematic overview of a low working memory load trial of the spatial span task (van Ewijk et al., 2015). In this example trial, a sequence of three (low load) yellow (working memory) circles was presented (500 ms per circle, with a 500 ms interstimulus interval; stimulus presentation). Next, a probe appeared, in this example prompting whether the second circle appeared in that position in the grid. Children were instructed to respond within a 2,000-ms response window, in this case responding with “yes” (i.e., the second circle was in that position). The response was followed by feedback (a red or green bar underneath the probe), which was presented for the remainder of the response window (response and feedback). In this example, a correct response (“yes”) was given, and a green bar appeared below the probe as feedback

An adapted version of a spatial span task developed by Klingberg, Forssberg, and Westerberg (2002) was used to assess VSWM (van Ewijk et al., 2014, 2015). The task was created in E-prime (version 2.0.10.356; Psychology Software Tools). A 4 × 4 grid was presented on a screen behind the MRI scanner that was visible for the child via a mirror attached to the head coil. In the grid, a sequence of either three (low working memory load) or five (high working memory load), either yellow (working memory condition) or red (control condition) circles was presented, 500-ms per circle, with an interstimulus interval of 500 ms (Fig. 1). Next, a probe was presented in one of the 16 possible locations in the grid, consisting of a number, referring to one of the presented stimuli, followed by a question mark. In the working memory conditions, children were instructed to remember the order in which the circles (three or five) were presented. When the probe was shown, the child had to indicate with a right (“yes”) or left (“no”) button press whether the probe location matched the location of the stimulus that was indicated by the probe number (see example in Fig. 1). Children were asked to respond within a 2,000-ms response window. In the control conditions, the circles (three or five) were shown in a predictable manner in the four corners of the grid and were always followed by a probe with the number 8. Children were instructed to look at the circles, but not to remember the order, and to always press “no” when the probe appeared. Feedback was provided in both conditions by presenting a green (correct response) or red (incorrect response) coloured bar underneath the probe. The task was administered in 4 blocks, each containing 24 trials, with a short break in between blocks, resulting in a total task duration of approximately 16 min. The percentage of the correct working memory trials (for the low and high working memory load trials separately, and for the low and high working memory load trials combined) were used as outcome measures for behavioral performance. Fig. 1 shows a schematic overview of the spatial span task.

#### Gross motor skills

Gross motor skills were evaluated using three subtests (jumping sideways, moving sideways, and backwards balancing) of the Korper Koordinationstest für Kinder (KTK) (Kiphard & Schilling, 2007). The KTK originally consists of four subtests, but a recent study has shown substantial agreement between the test battery consisting of three subtests and the original test battery consisting of four subtests (Novak et al., 2017). Additionally, one item of the Bruininks-Oseretsky Test of Motor Proficiency, Second Edition (BOT-2; Bruininks & Bruininks, 2005) was used to measure ball skills. Both test batteries have shown to be reliable and valid for primary school children (Bruininks, 2005; Bruininks & Bruininks, 2005; Kiphard & Schilling, 2007; Novak et al., 2017).

##### Jumping sideways (KTK)

Children jumped laterally as quickly as possible over a small wooden slat (60 × 4 × 2 cm) for 15 s. The total number of jumps in two trials was used as the score for jumping sideways.

##### Moving sideways (KTK)

Children moved across the floor as quickly as possible in 20 s by stepping on, and transferring two plates (25 × 25 × 5.7 cm). Children stepped from the first plate to the next, subsequently lifting and transferring the first plate alongside the second and stepping on it. Each successful transfer from one plate to the next resulted in two points: one for shifting the plate and one for stepping on the next plate. The total number of points on two trials was used as a score for moving sideways.

##### Backwards balancing (KTK)

Children made as many steps backwards as possible on three wooden beams with lengths of 3 m, but decreasing in width (resp. 6 cm, 4.5 cm, and 3 cm). For each beam, children performed three trials. A maximum of eight steps per trial was counted, resulting in a maximum score of 72.

##### Ball skills (BOT-2)

The ball skills subtest consisted of seven activities executed with a tennis ball. Activities were catching, throwing and dribbling a ball with one or both hands and throwing a ball at a target. For each task, five or seven trials were performed. For each correct trial, a child received one point, resulting in a maximum score of 39 points.

#### Cardiovascular fitness

Cardiovascular fitness was administered with the 20-meter Shuttle Run Test (20-m SRT, in number of completed stages; Adam, Klissouras, Ravazzolo, Renson, & Tuxworth, 1988). In the 20-m SRT, children run back and forth over a distance of 20 m, indicated by lines on the floor. An audio signal sounds at the moment in time that children must have covered the distance on the track by touching the line with one of their feet. The required average speed to cover the track is initially set at 8 km/h and increases every minute by 0.5 km/h. The test was terminated for a child when he/she failed to reach the other end of the track in time on two consecutive occasions. Validity and reliability of the SRT have shown to be adequate in children (Leger, Mercier, Gadoury, & Lambert, 1988).

### Procedure

Data were collected during school year 2016/2017. VSWM was assessed during a functional MRI scan, carried out as part of a scanning protocol that was performed at Vrije Universiteit Medical Centre in Amsterdam (n = 47), or at the University Medical Center in Groningen (n = 45). Children were familiarized with the scanning procedure using a mock scanner and with the task in a half hour session before data collection. Children responded to the task using a button-box (Current designs Inc., Philadelphia, PA). Head movements were minimized by inserting small, wedge-formed pillows between the head coil and the child’s head. Children received a small present and a copy of their structural T1-weighted scan.

Cardiovascular fitness and motor skills were assessed by trained research assistants using standardized protocols, at the children’s own school, within a time frame of 2 weeks around the scanning procedure. Cardiovascular fitness was assessed during a physical education lesson in groups of up to 15 children. Motor skills were individually assessed during one or two (depending on the class size) physical education lessons, in circuit form, with tests administered in a random order.

### Image acquisition

The imaging protocol was carried out at two different sites (Amsterdam and Groningen) on either a 3 Tesla whole-body unit (Discovery MR750, GE Healthcare, Milwaukee, WI; Amsterdam) or a 3 Tesla Philips Intera scanner (Philips Medical Systems, Best, the Netherlands; Groningen), using a 32-channel head coil and closely-matched acquisition parameters. Blood-oxygen-level-dependent (BOLD) contrasts with T2*-weighted functional gradient echo-planar images (EPI) were obtained using the following parameters: repetition time (TR) = 2,000 ms, echo time (TE) = 35 ms, flip angle (FA) = 80°, field of view (FOV) = 211 mm, slice thickness = 3.0 mm, interslice distance = 0.3 mm, 135 dynamics, and 64 × 64 grid (Amsterdam protocol), or 64 × 60 grid (Groningen protocol), voxel size = 3.3 × 3.3 × 3.3 mm. Four runs were obtained. Two spin echo EPI scans with opposing polarities of the phase-encode blips were acquired (TR = 6,000 ms, TE = 60 ms, all other parameters remained the same), which would later be applied to correct for distortions in the functional images caused by the susceptibility distribution of the subjects head (Andersson & Sotiropoulos, 2016; Smith et al., 2004). Additionally, high-resolution, whole-brain, T1-weighted sagittal brain images were acquired at the beginning of the scan protocol (TR = 400 ms, TE = min full, FA = 111°, FOV = 250 mm, slice thickness = 3.0 mm, interslice distance = 0.3 mm, and 256 × 192 grid, voxel size = 1 × 1 × 1 mm).

### Image analyses

#### First level analysis

For each subject, data were preprocessed using FLS feat (FMRI Expert Analysis Tool; FMRIB Analysis group, Oxford, UK; available from the FMRIB Software Library at www.fmrib.ox.ac.uk/fsl). The first steps (until the data were combined into a single 4D dataset) were performed separately for all the four experimental blocks. Blocks were only included if (1) there was at least one correct response for each of the four conditions (working memory and control conditions, high and low memory load), and (2) the block was complete, i.e., the scan was not aborted before the end of the block. In total, 91.3% of the blocks was included in the analyses. Functional images were corrected for head motion using rigid body transformations (MCFLIRT, FSL; Jenkinson, Bannister, Brady, & Smith, 2002), followed by a correction for the susceptibility distribution of the subjects head (TOPUP tool in FSL; Andersson & Sotiropoulos, 2016; Smith et al., 2004). To remove non-brain tissue from the functional scans and the T1-weighted structural images, the Brain Extraction Tool (BET; Smith, 2002) was applied. Subsequently, spatial smoothing was applied to the functional data using a 5-mm Full Width Half Maximum (FWHM) Gaussian Kernel. Smoothing was applied to improve signal-to-noise ratio by replacing the value of a single voxel by a weighted average of neighboring voxels. Finally, the experimental blocks were combined into a single 4D dataset per subject, which could be used for further analyses.

To remove artefacts from the subject’s data, an independent-component analysis (ICA) was conducted using Multivariate Exploratory Linear Optimized Decomposition into Independent Components (MELODIC; Beckmann & Smith, 2005) for each subject’s 4D dataset. MELODIC is a method by which a 4D dataset can be decomposed into spatial and temporal components. This way, activation and artefactual components can be distinguished, because they have unique spatial patterns (Kelly et al., 2010; Thomas, Harshman, & Menon, 2002). Based on the recommendation to use about one-fourth to one-fifth of the total of time points in the scans (Greicius, Srivastava, Reiss, & Menon, 2004), and previously widely adopted settings of 20-30 components for ICA (Smith et al., 2009), a fixed number of 30 components was extracted per subject. The spatial component maps were visually inspected for artefacts, and components representing artefacts were removed. A description of the ICA analysis procedure can be found in Appendix 1.

The remaining components were used to generate contrast images using Statistical Parameter Mapping 12.0 (SPM 12.0 v6470, running in MATLAB 2017b). The task-conditions are presented in Table 1. Only correct trials were included to minimize variability in brain activation between different conditions, because differences in brain activation were expected during incorrect and omission trials compared with correct trials. Two contrast images were constructed per subject, representing differences in brain activation for each voxel when comparing different conditions: a working memory contrast (successful working memory trials [Con1 and Con4] versus successful control trials [Con7 and Con10]) and a load difference contrast (successful high working memory load trials [Con4] versus successful low working memory load trials [Con1]). The contrast images were rescaled by dividing their intensity scale by its respective standard deviation, because a difference between the two sites was found in the scaling of the contrast images. The resulting contrast images were coregistered to the subject’s own 3D anatomical space, normalized using an MNI-152 template, and smoothed with an 8-mm FWHM Gaussian Kernel in SPM 12.0.

**Table 1:** Overview of all task-conditions in the Visuospatial Working Memory Task

	Working memory trials		Control trials	
	Low load	High load	Low load	High load
Correct response	*Con1*	*Con4*	*Con7*	*Con10*
Incorrect response	Con2	Con5	Con8	Con11
Omission error	Con3Con6	Con9	Con12	

Conditions used for this study (only correct trials) are shown in italics

*Con* condition

Children were excluded from the analysis if (1) more than 15 components were manually removed from the data (*n* = 3); (2) normalization had failed (*n* = 7), or (3) they were absent on testing days at school, and therefore had no score for cardiovascular fitness and/or motor skills (*n* = 1). An overview of the number of children that participated (separated by school, grade, and sex), and the final number of children that was included for the data analyses is presented in Appendix 2. The final sample consisted of 80 children (87.0% of the total number of children that was scanned: 41 girls [51.3%]; 38 grade 3 children [47.5%]).

### Statistical analysis

#### Behavioral data

A principal component analysis on the standardized scores of the gross motor skill tests was performed to calculate a Bartlett factor score. This analysis was performed on the total sample of 891 children in the Learning by Moving study (see van der Fels et al., 2019)*.* The four gross motor skill components loaded highly (>0.6) onto one factor and explained 48.2% of the total variance. This factor was used in the analysis as a measure of gross motor skills.

IBM SPSS Statistics version 25.0 was used to calculate Pearson correlations between the physical task scores (gross motor skills and cardiovascular fitness) and behavioral VSWM task scores (low working memory load trials, high working memory load trials, and low and high working memory trials together) for the children who participated in this fMRI study. Level of significance was set at *p* < 0.05.

#### Second level fMRI analysis

The fMRI data were analyzed in SPM12.0. In a first step, two General Linear Models (GLM) were created (one for each contrast) to capture the overall BOLD response. The contrast images from the first level analysis were added as dependent variable in the models. Additionally, scan site (Amsterdam or Groningen), sex, age, and SES were included in the model as covariates of no interest. In a second step, a GLM was created for both contrasts with the factor score for gross motor skills as covariate of interest. Finally, a GLM was created for both contrasts with cardiovascular fitness as covariate of interest. If the covariates of no interest included in step 1 were significant, they were included in the models created in steps 2 and 3 as well. Figures shown in this article represent activation maps with a threshold at significance level of *p* < 0.01 (uncorrected). Tables and text in the results section will represent results that survived the cluster level significance of *p* < 0.05, family wise error (FWE) corrected, initial threshold *p* < 0.001.

## Results

### Behavioral results

Demographics and scores on cardiovascular fitness, gross motor skills, and VSWM are shown in Table 2. Pearson correlations showed that gross motor skills were positively related to task performance on low working memory load trials, *r* = 0.364, *p* = 0.001, to high working memory load trials, *r* = 0.236, *p* = 0.035, and to all working memory trials, *r* = 0.322, *p* = 0.004. Cardiovascular fitness also was positively related to task performance on low working memory load trials, *r* = 0.279, *p* = 0.012, to high working memory load trials, *r* = 0.221, *p* = 0.049, and to all working memory trials, *r* = 0.268, *p* = 0.016.

**Table 2:** Pearson correlations between the study variables, and descriptive statistics and test scores (means and standard deviations) of the total sample (*n* = 80)

	Age (yr) ^a^	Sex (% girls)	SES ^a^	Grade (% grade 3)	Low VSWM load trials (% correct)^a^	High VSWM load trials (% correct)^a^	All VSWM trials (% correct)^a^	Gross motor skills (factor score)^a^	Cardiovascular fitness (stages)^a^
Age (yr)^a^	1								
Sex (% girls)	-0.046	1							
SES^a^	-0.081	0.022	1						
Grade (% grade 3)	0.796**	0.074	0.056	1					
Low VSWM load trials (% correct)^a^	0.048	-0.061	0.207	0.160	1				
High VSWM load trials (% correct)^a^	-0.035	0.025	0.224*	0.093	0.750**	1			
All VSWM trials (% correct)^a^	0.008	-0.020	0.23*	0.136	0.937**	0.934**	1		
Gross motor skills (factor score)^a^	0.268*	-0.057	0.020	0.306**	0.364**	0.236*	0.322**	1	
Cardiovascular fitness (stages)^a^	0.198	-0.257*	0.154	0.142	0.279*	0.221*	0.268*	0.494**	1
Mean (*SD*) or percentage	9.17 (0.62)	51.30	4.58 (1.06)	47.50	70.70 (15.97)	66.00 (15.54)	68.35 (14.74)	0.18 (1.01)	4.74 (1.91)

*Note.* Performance on low and high working memory significantly differed as measured with a paired sample *t*-test, *t* (80) = 4.245, *p* < 0.001; SES = socioeconomic status, obtained by a parental questionnaire. Level of parental education of both parents was requested and varied from 0 (no education) to 7 (postdoctoral education; Schaart, Mies, & Westerman, 2008). Average education level of both parents was used as a measure of SES. If the level of parental education was specified for only one of the parents, this level was used as a measure of SES for the child

^a^Mean (SD); * *p* < 0.05; ** *p* < 0.01

### fMRI results

#### Working memory contrast

**Fig. 2:**
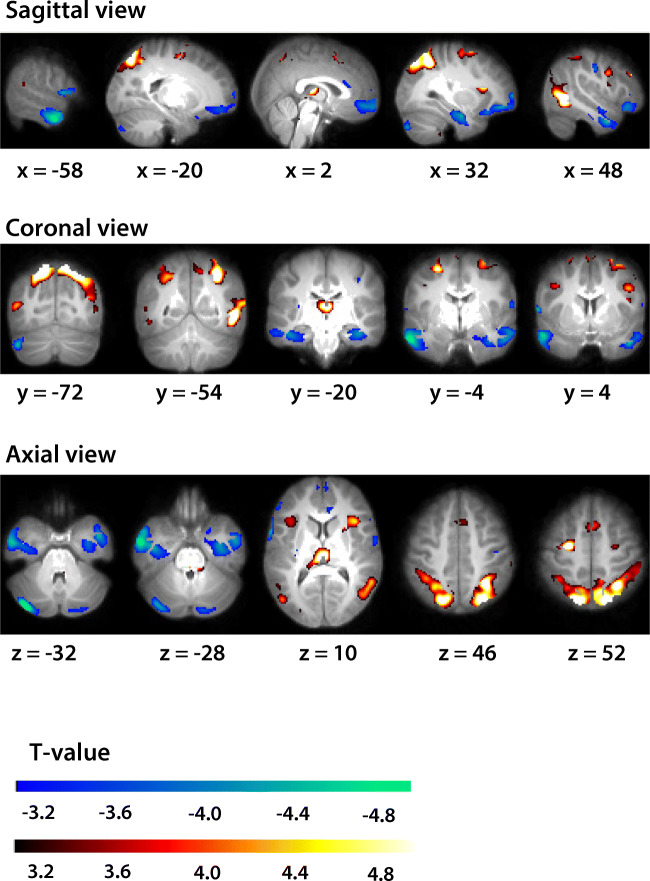
Brain activation for the working memory contrast. Axial (upper), coronal (middle), and sagittal (lower) view. Warm colours indicate activation in working memory trials as compared to control trials. Cool colours indicate deactivation in working memory trials as compared to control trials. MNI coordinates (x, y, and z) represent the location of the maximum intensity voxel

Brain activation during working memory trials compared to control trials, while controlling for the covariates of no interest that were included in step 1 (i.e., scan site, sex, age, and SES) are shown in Fig. 2. Table 3 shows MNI coordinates of the significant clusters of brain activation. Significant clusters were located right in the angular gyrus and bilateral in the superior parietal cortex, the inferior temporal gyrus, and the middle temporal gyrus (*p* < 0.05), indicating task related increases in activation in the angular and superior parietal areas, and task related decreases in the inferior and middle temporal areas. Results on the covariates (scan site, age, sex, and SES) are presented in Appendix 3. Only scan site was a significant covariate and was therefore included as a covariate of no interest in the all subsequent analyses.

**Table 3:** Significant clusters of brain activation associated with visuospatial working memory, controlling for scan site, age, sex, and SES

Cluster	Anatomical label(s)	Hemisphere	N voxels	MNI coordinates		
				X	Y	Z
1	Angular gyrus, superior parietal gyrus^a^	Right	3900	32	−54	46
2	Superior parietal gyrus^a^	Left	1562	−20	−72	52
3	Thalamus^a^	Bilateral	503	2	−20	10
4	Inferior temporal gyrus, middle temporal gyrus^b^	Left	6940	−58	−4	−28
5	Inferior temporal gyrus, middle temporal gyrus^b^	Right	1498	48	4	−32

*Note*: Activation for the working memory contrast that survived the cluster level significance of *p* < 0.05, family wise error (FWE) corrected, initial threshold *p* < 0.001. N voxels: number of voxels involved in the significant cluster (total brain volume consisted of 153138 voxels). MNI coordinates represent the location of the maximum intensity voxel

^a^Brain areas indicating activation in working memory trials as compared to control trials

^b^Brain areas indicating deactivation in working memory trials as compared to control trials

#### Load difference contrast

Although the percentage of correct trials was higher for low working memory (70.7 %) load than for high working memory load (66.0 %; *p* < 0.01), analysis on the load difference contrast revealed no significant differences in activation between high and low working memory load (all *p* > 0.05). Therefore, this contrast was not further examined.

#### Gross motor skills and cardiovascular fitness

The results regarding gross motor skills and cardiovascular fitness revealed no significant associations between either gross motor skills or cardiovascular fitness with brain activation (*p* > 0.05), indicating that both gross motor skills and cardiovascular fitness were not related to VSWM-related brain activation.

## Discussion

The main purpose of this study was to investigate the relationship of gross motor skills and cardiovascular fitness with VSWM-related brain activation in 8- to 10-year-old typically developing children. VSWM-related brain activation was found in a neural network involving the angular gyrus (right hemisphere), the superior parietal cortex (bilateral), and the thalamus (bilateral). In addition, VSWM-related deactivation was found in the inferior and middle temporal gyri (bilateral). Gross motor skills and cardiovascular fitness were not associated with VSWM-related brain activation, while there were significant relations of gross motor skills and cardiovascular fitness with behavioral VSWM performance. Therefore, we could not confirm the hypothesis that functional brain mechanisms underlie the relations of gross motor skills and cardiovascular fitness with VSWM in 8- to 10-year-old children.

### VSWM-related brain activation patterns

The brain regions that were found to be involved in VSWM task performance are partly in accordance with brain regions found to be associated with VSWM in the literature. As summarized in a meta-analysis by Wager and Smith (2003), spatial storage tasks most frequently activate the superior parietal cortex, which also was found in our study. Furthermore, it has been shown that during visuospatial working memory tasks, the prefrontal cortex is interconnected with posterior parietal and temporal cortices and with subcortical areas, such as the thalamus (van Ewijk et al., 2015; Klingberg et al., 2002; Goldman-Rakic, 2011), an area where we found VSWM-related activation as well. However, contradicting these previous findings, we found deactivation in the inferior and middle temporal gyrus, and there was no difference in activation in prefrontal areas. It is difficult to explain these findings, because previous studies in children have constantly found increased activation in working memory trials compared with control trials in temporal and prefrontal areas, based on which it is expected that working memory trials require more brain activation than control trials (van Ewijk et al., 2015; Klingberg et al., 2002).

There were no differences in brain activation between the high working memory load trials and the low working memory load trials, although children performed significantly better on low working memory load trials than on high working memory load trials. This was unexpected based on a previous study by van Ewijk et al. (2015) in which the same task was used. In their study, participants also performed better on low working memory load trials (75% correct) than on high working memory load trials (80% correct), but this was related to differences in brain activation in the frontal, temporal, occipital, and parietal regions. In the current study, participants performed worse on both high working memory load trials (66% correct) and low working memory load trials (71% correct) than the participants in the study by van Ewijk et al. (2015). Possibly, performance levels of the children in the current study on both high and low working memory load trials were not stable enough, and therefore, there were no differences in brain activity between the high and low working memory load trials. Furthermore, the power in our study might have been too low to detect differences in brain activation compared with the study by van Ewijk et al. (2015), who included a much larger sample (*N* = 212).

### Relationship with gross motor skills and cardiovascular fitness

Neither gross motor skills nor cardiovascular fitness was related to the neural network supporting VSWM. Although both gross motor skills and cardiovascular fitness were significantly related to behavioral VSWM performance, we could not confirm the hypothesis that the neural network supporting VSWM underlies the relationship of gross motor skills and cardiovascular fitness with VSWM. Our results are contradictory to the studies by Chaddock et al. (2012) and Voss et al. (2011), where associations between cardiovascular fitness and brain activation were found. In those studies, cardiovascular fitness was measured by estimating the VO_2_ max of children during a running test on a treadmill, whereas in the current study, cardiovascular fitness was assessed with the 20-m SRT. The estimation of the VO_2_ max in the studies by Chaddock et al. (2012) and Voss et al. (2011) was possibly more sensitive in measuring differences in cardiovascular fitness level than the 20-m SRT, which might have been a reason that we did not find associations between cardiovascular fitness and VSWM-related brain activity. Furthermore, it should be noted that Chaddock et al. (2012) and Voss et al. (2011) measured brain activation during an inhibition task. A review by Haapala (2013) revealed that physical fitness and gross motor skills were differently related to specific cognitive functions. Possibly then, the relationship of cardiovascular fitness and gross motor skills with executive functioning-related brain activity differ depending on the specific executive function being examined (i.e., inhibition, working memory, or cognitive flexibility). For future studies, it would be interesting to compare relations of gross motor skills and cardiovascular fitness with brain activity patterns underlying the different executive functions.

### Strengths, limitations, and future directions

Strengths of this study include the large sample of typically developing children that was examined. Previous studies on the relationships between physical fitness variables and brain functioning used sample sizes varying from 36–52 children (Chaddock et al., 2012; Voss et al., 2011; Ludyga et al., 2018, 2019). We analyzed data from 80 children. This enabled us to get a detailed and reliable insight in brain activation during a VSWM task. Additionally, by including both gross motor skills and cardiovascular fitness it was possible to examine underlying brain mechanisms in the relations of gross motor skills and cardiovascular fitness with VSWM performance.

However, this study also showed that it is difficult to perform a (f)MRI study in young children, because participating children had difficulties with laying still throughout the scanning protocol. The total acquisition protocol had a total scan time of approximately 1 h. The active state scan used for this study was the last part of the protocol, which explains why it was difficult for children to remain still, resulting in movement artefacts in the fMRI data. By applying extensive preprocessing steps, we tried to minimize the effect of these movement artefacts. Still, subtle changes in brain activity related to gross motor skills and/or cardiovascular fitness might have been filtered out by the preprocessing steps that we applied.

Furthermore, factors such as sleep, experienced disturbances throughout the day, tiredness, and moment of testing could have influenced VSWM performance and VSWM-related brain functioning (Dirk & Schmiedek, 2016, 2017; Könen, Dirk, & Schmiedek, 2015). We did not control for such confounding variables in our analyses, which could have influenced the outcome of our study. For future studies, it is recommended to control for factors that can influence VSMW performance and VSWM related brain activity.

Our results might further be limited by the way that we represented children’s gross motor skills. The total factor score that we calculated explained only 48.2% of the variance of the gross motor skill scores. Therefore, we could have missed aspects of gross motor skills that might have been related to VSWM-related brain activity. Furthermore, the review by van der Fels et al. (2015) showed that the strongest relationships are found between complex motor skills (e.g., fine motor skills or bilateral body coordination) and executive functions. Circuit-based assessments have recently emerged as a dynamic method and more complex way of measuring motor skills. These tests are more sensitive to assessor experience, however, and the validity and reliability of circuit-based assessments need to be further investigated (Robinson et al., 2015). At the neuropsychological level, it can be argued that these complex motor skills require greater involvement of executive functions than relatively simple motor skills (Best, 2010; van der Fels et al., 2015). This implies that complex forms of motor skills share more overlapping neural networks with executive functions than gross motor skills. For future studies, it would be interesting to use tests that measure more complex forms of motor skills than the BOT-2 and KTK do.

Although the cross-sectional nature of our study does not allow us to draw causal inferences, our results suggest that improving children’s cardiovascular fitness or motor skills will not necessarily result in changes in brain activity. Yet, several studies have shown that physical activity interventions can result in changes in brain structure and function, going hand in hand with improvements in cognitive functioning (Davis et al., 2011; Hillman et al., 2014; Krafft et al., 2014; Gunnell et al., 2019). Because these studies did not examine changes in cardiovascular fitness or motor skills, it would be interesting to include these measures in future research at well. This will give a better idea of direct and indirect relationships among gross motor skills, cardiovascular fitness, VSWM-related brain activity, and cognitive performance.

In addition, the current study examined whether functional brain activity patterns measured with the BOLD signal underlie the relationships of gross motor skills and cardiovascular fitness with VSWM. We did not find support for this hypothesis. It is therefore questionable whether brain activation patterns measured with the BOLD signal are the best way to investigate the mechanisms underlying the relationships of gross motor skills and cardiovascular fitness with VSWM. Possibly, imaging techniques that measure structural connectivity of white matter or functional connectivity will give a better insight in the brain mechanisms underlying the relationships of gross motor skills and cardiovascular fitness with VSWM.

## Conclusions

Regions in the parietal and temporal cortices and the thalamus were found to be important for VSWM performance in 8- to 10-year-old children. Activation patterns did not differ between high and low working memory load trials. Although gross motor skills and cardiovascular fitness were both related to VSWM performance, they were not related to VSWM-related brain activation. Based on these results, we could not confirm the hypothesis that brain activation patterns underlie the relationship of gross motor skills and cardiovascular fitness with VSWM performance. Our results suggest that higher levels of cardiovascular fitness or gross motor skills will not necessarily result in changes in brain activity. Because previous interventional studies have not yet related changes in brain activity and cognition to changes in gross motor skills or cardiovascular fitness, further research is needed to get a better idea of the direct and indirect relationships among gross motor skills, cardiovascular fitness, cognitive functioning, and underlying brain changes. Our results suggest that brain activation patterns as measured with the BOLD signal may not be the best way to examine the mechanism underlying the relationships of gross motor skills and cardiovascular fitness with VSWM. Further research should use imaging techniques that measure structural and functional connectivity to investigate the mechanisms underlying the relationships of gross motor skills and cardiovascular fitness wilt VSWM.

## Figures and Tables

**Figure:**
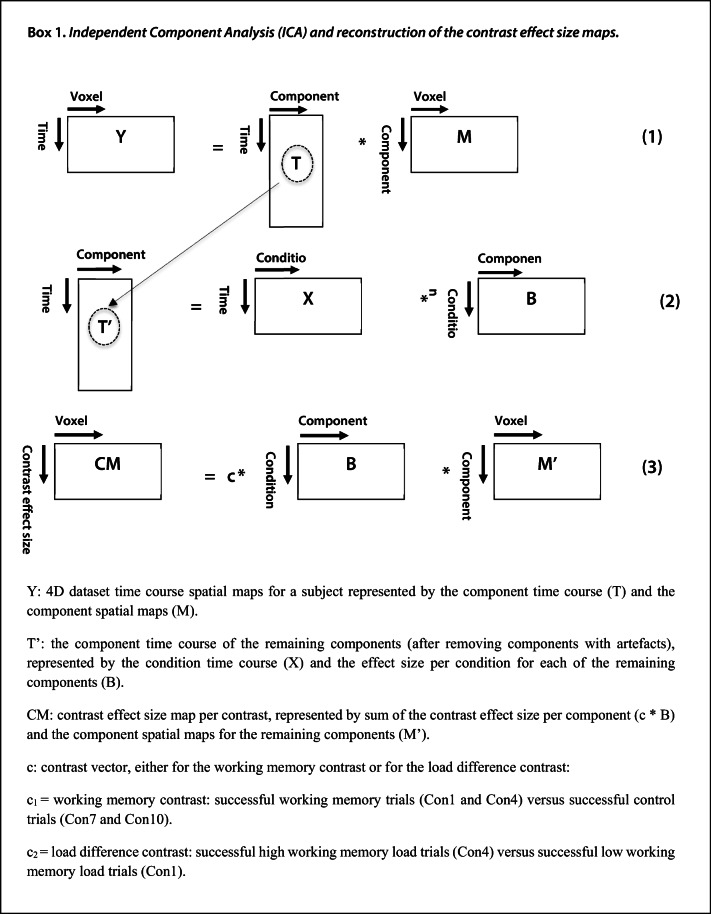
Box 1. Independent Component Analysis (ICA) and reconstruction of the contrast effect size maps

**Fig. 3:**
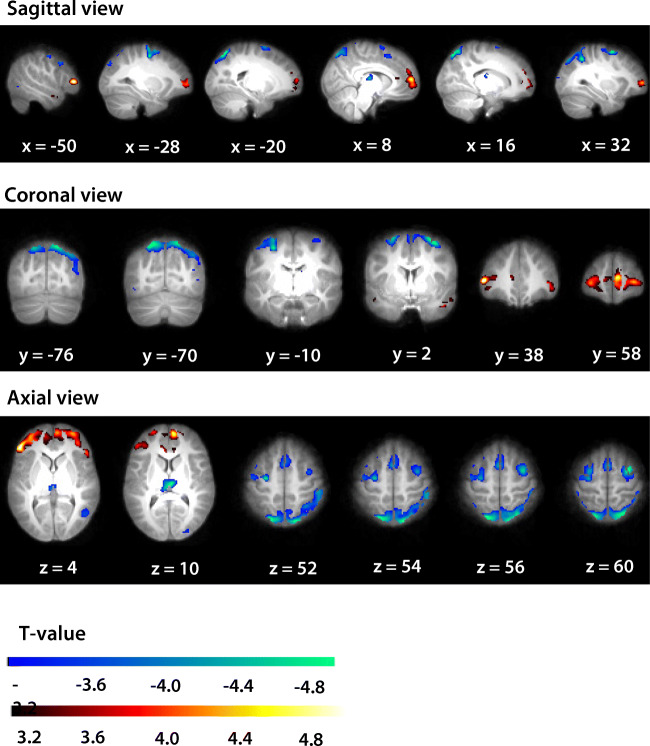
Difference in brain activation between children scanned in Amsterdam and in Groningen. Axial (upper), coronal (middle), and sagittal view (lower). Threshold is set at *p* < 0.001 (uncorrected). Warm colours indicate activation in children scanned in Amsterdam as compared to children scanned in Groningen. Cool colours indicate deactivation in children scanned in Amsterdam compared with children scanned in Groningen. MNI coordinates (x, y, z) represent the location of the maximum intensity voxel

## Appendix 1. Description of the ICA analysis steps

To remove artefacts from the subject’s data, an independent-component analysis (ICA) was conducted by using Multivariate Exploratory Linear Optimized Decomposition into Independent Components (MELODIC; Beckmann & Smith, 2005) for each subject’s 4D dataset. MELODIC is a method by which a 4D dataset can be decomposed into spatial and temporal components. This way, activation and artefactual components can be distinguished, because they have unique spatial patterns (Kelly et al., 2010; Thomas et al., 2002). By using ICA, the data were represented by a multiplication of two matrices (see Box 1):

1 $$ \mathrm{Y}=\mathrm{T}\ast \mathrm{M}; $$

in which Y represents the time course spatial maps (dimension time by voxel), T represents the component time course (dimension time by component), and M the component spatial maps (component by voxel). Based on the recommendation to use about one-fourth to one-fifth of the total of time points in the scans (Greicius et al., 2004), and previously widely adopted settings of 20-30 components for ICA (Smith et al., 2009), a fixed number of 30 components was extracted per subject. The spatial component maps were visually inspected for artefacts, and components representing artefacts were removed. The remaining components (T’, M’) were used to generate contrast images (i.e., a representation of differences in brain activation between different task conditions), using the following procedure:

1. A model representing the expected blood-oxygen-level-dependent (BOLD) response was created for each of the task-conditions (X: dimension time by condition) using Statistical Parameter Mapping 12.0 (SPM 12.0 v6470, running in MATLAB 2017b). The task-conditions are presented in Table 4. Only correct trials were included to minimize variability in brain activation between different conditions, because differences in brain activation were expected during incorrect and omission trials compared with correct trials. The model was created by convolving a stick function with a canonical Hemodynamic Response Function (HRF). Additionally, a constant was added to this model to capture an offset.
2. The time course of each of the remaining components T′ was regressed against the model created in step 1 using Ordinary Least Square (OLS), resulting in an effect size per condition (B: dimension condition by component):

**Table 4:** Overview of all task-conditions in the Visuospatial Working Memory Task

	Working memory trials		Control trials	
	Low load	High load	Low load	High load
Correct response	*Con1*	*Con4*	*Con7*	*Con10*
Incorrect response	Con2	Con5	Con8	Con11
Omission error	Con3	Con6	Con9	Con12

Conditions used for this study (only correct trials) are shown in italics

*Con* condition

2 $$ \mathrm{T}^{\prime }=\mathrm{X}\ast \mathrm{B} $$

3) Two contrast vectors (c_1_, c_2_) were defined in order to reconstruct a contrast effect size map per subject (CM: dimension contrast effect size by voxel), representing differences in brain activation when comparing different conditions:

- A working memory contrast (c_1_): successful working memory trials (Con1 and Con4) versus successful control trials (Con7 and Con10);
- A load difference contrast (c_2_): successful high working memory load trials (Con4) versus successful low working memory load trials (Con1).

For each voxel in CM, the contrast effect size was reconstructed by summing the contrast effect size per component (c*B) across components, weighted by the corresponding value in M′. This way, components with larger effect sizes had a higher weight in reconstruction of the maps:

3 $$ \mathrm{CM}=\mathrm{c}\ast \mathrm{B}\ast \mathrm{M} $$

This resulted in a contrast image representing the activation differences between the conditions for each voxel per subject. A difference between the two sites was found in the scaling of the contrast-images, as the intensity scale of the images acquired in Groningen was five times larger than that of those acquired in Amsterdam. The images were therefore rescaled by dividing their intensity scale by its respective standard deviation.

4) The contrast image CM was coregistered to the subject’s own 3D anatomical space and normalized to standard space by registration to an MNI-152 template. Normalization is used in order to match anatomical brain locations across subjects. This allows averaging brain activation patterns across subjects and therefore can be used for further second level (group) analysis. The contrast CM images were spatially smoothed with an 8-mm FWHM Gaussian Kernel. The smoothing, co-registration and normalization steps were performed in SPM.

## Appendix 2. Inclusion protocol

This table shows the number of children per grade/sex/scan site that were planned to be scanned, that were actually scanned and that were used for analyses.

**Table 5:** Number of children planned, scanned, and analyzed, per site, grade, and sex

	Grade 3	Grade 4	Total
Boys			
Planned	23	22	45
Scanned	24	21	45
Analyzed	20	19	39
Amsterdam planned	12	10	22
Amsterdam scanned	13	10	23
Amsterdam analyzed	11	10	21
Groningen planned	11	12	23
Groningen scanned	11	11	22
Groningen analyzed	9	9	18
Girls			
Planned	22	23	45
Scanned	22	25	47
Analyzed	18	23	41
Amsterdam planned	12	11	23
Amsterdam scanned	12	12	24
Amsterdam analyzed	11	12	23
Groningen planned	10	12	22
Groningen scanned	10	13	23
Groningen analyzed	7	11	18
Total			
Planned	45	45	90
Scanned	46	46	92
Analyzed	38	42	80
Amsterdam planned	24	21	45
Amsterdam scanned	25	22	47
Amsterdam analyzed	22	22	44
Groningen planned	21	24	45
Groningen scanned	21	24	45
Groningen analyzed	16	20	36

## Appendix 3. Results of the contribution of the covariates to VSWM-related brain activation

Age, sex, and SES did not contribute significantly to VSWM-related brain activation (*p* > 0.05). However, there was a significant difference between brain activation of children scanned in Amsterdam and those who were scanned in Groningen (Fig. 3), located bilateral in superior parietal gyrus and the anterior prefrontal gyrus, bilateral in the premotor and supplementary motor cortex, and left in the angular gyrus and the inferior frontal gyrus. Scan site was included as covariate in all analyses.

**Table 6:** Significant clusters of brain activation associated with scan site

Cluster #	Anatomical label(s)	Hemisphere	N voxels	MNI coordinates^a^		
				X	Y	Z
	Working memory contrast					
1	Superior parietal gyrus, angular gyrus^b^	Left	739	−20	−70	56
2	Superior parietal gyrus^b^	Right	1907	16	−76	54
3	Premotor cortex, supplementary motor cortex^b^	Right	725	32	2	60
4	Premotor cortex, supplementary motor cortex^b^	Left	601	−28	−10	52
5	Inferior frontal gyrus and anterior frontal gyrus	Left	829	−50	38	4
6	Anterior prefrontal gyrus	Right	1429	8	58	10

*Note*: Activation for the working memory contrast that survived the cluster level significance of *p* < 0.05, family wise error (FWE) corrected, initial threshold *p* < 0.001. N voxels: number of voxels involved in the significant cluster (total brain volume consisted of 153138 voxels)

^a^Brain coordinates defined by the Montreal Neurological Institute (MNI), based on which the location of (de)activated clusters of voxels can be identified. MNI coordinates represent the location of the maximum intensity voxel

^b^Brain areas indicating deactivation in children scanned in Amsterdam compared with children scanned in Groningen
